# Efficacy and Drug Survival after Switching from Etanercept to the Biosimilar SB4: A Real-Life Long-Term Study

**DOI:** 10.3390/jcm11030621

**Published:** 2022-01-26

**Authors:** Simone Parisi, Andrea Becciolini, Maria Chiara Ditto, Davide Rozza, Anna Zanetti, Angela Laganà, Clara Lisa Peroni, Chiara Centanaro Di Vittorio, Rosanna Degiovanni, Cristina Realmuto, Carlo Alberto Scirè, Marta Priora, Eleonora Di Donato, Daniele Santilli, Flavio Mozzani, Gianluca Lucchini, Alarico Ariani, Lucia Gardelli, Francesco Girelli, Eugenio Arrigoni, Ilaria Platè, Elena Bravi, Marino Paroli, Rosalba Caccavale, Carlo Salvarani, Gilda Sandri, Federica Lumetti, Alessandro Volpe, Antonio Marchetta, Enrico Fusaro

**Affiliations:** 1 Rheumatology Unit, Department of General and Specialistic Medicine, Azienda Ospedaliero-Universitaria, Città della Salute e della Scienza di Torino, 10121 Turin, Italy; mariachiaraditto@gmail.com (M.C.D.); alagana2@cittadellasalute.to.it (A.L.); cperoni@cittadellasalute.to.it (C.L.P.); ccentanarodivittorio@cittadellasalute.to.it (C.C.D.V.); rdegiovanni@cittadellasalute.to.it (R.D.); cristina.realmuto@gmail.com (C.R.); fusaro.reumatorino@gmail.com (E.F.); 2 Internal Medicine and Rheumatology Unit, Department of Medicine, Azienda Ospedaliero-Universitaria di Parma, 43121 Parma, Italy; beccio@yahoo.com (A.B.); eleonoradidonato@ymail.com (E.D.D.); dsantilli@ao.pr.it (D.S.); fmozzani@ao.pr.it (F.M.); gianluca.lucchini76@gmail.com (G.L.); atendoro@gmail.com (A.A.); 3 Epidemiology Research Unit, Italian Society for Rheumatology, 20019 Milan, Italy; d.rozza@reumatologia.it (D.R.); a.zanetti@reumatologia.it (A.Z.); c.scire@reumatologia.it (C.A.S.); 4 Department of General and Specialist Medicine, Rheumatology Clinic, Hospital of Mondovì, 12100 Cuneo, Italy; marta.priora@gmail.com; 5 Internal Medicine Unit, GB Morgagni Hospital, 47121 Forli, Italy; lucia.gardelli@auslromagna.it (L.G.); fgirodoc@gmail.com (F.G.); 6 Department of Rheumatology, Ospedale Guglielmo da Saliceto, 29121 Piacenza, Italy; e.arrigoni@ausl.pc.it (E.A.); ilariaplate@gmail.com (I.P.); bravielena@yahoo.it (E.B.); 7 Department of Medical-Surgical Sciences and Biotechnologies, Sapienza University of Rome, Polo Pontino, 04100 Latina, Italy; marino.paroli@uniroma1.it (M.P.); rosalba_caccavale@yahoo.it (R.C.); 8 Rheumatology Unit, University of Modena and Reggio Emilia, 41100 Modena, Italy; carlo.salvarani@unimore.it (C.S.); gilda.sandri@unimore.it (G.S.); 9 Rheumatology Unit, Azienda USL of Modena and AOU Policlinico of Modena, 41100 Modena, Italy; fedelumetti@gmail.com; 10 Rheumatology Unit, IRCCS Sacro Cuore Don Calabria, 37024 Negrar, Italy; alessandro.volpe@sacrocuore.it (A.V.); antonio.marchetta@sacrocuore.it (A.M.)

**Keywords:** inflammatory arthritis, anti-TNF, biosimilar, SB4, drug survival

## Abstract

We evaluated the 3-year drug survival and efficacy of the biosimilar SB4/Benepali in rheumatoid arthritis (RA), psoriatic arthritis (PsA) and ankylosing spondylitis (AS) patients, previously treated with etanercept (ETA). Drug survival rate was calculated using the Kaplan–Meier method and Cox proportional hazard models were developed to examine predictors of SB4 discontinuation. 236 patients (120 RA, 80 PsA and 36 AS), aged 60.7 ± 13.8 years and with an ETA duration of 4.1 ± 3.4 years were included. The 3-year retention rate for SB4 was 94.4%, 88% and 86% in AS, RA and PsA patients, respectively, with no difference between groups. Patients without comorbid disease had higher retention rates vs. patients with comorbid disease (90% vs. 60%, *p* < 0.0001). Disease activity, as measured by DAS28, DAPSA and BASDAI remained stable over the 3 years. Comorbid disease (hazard ratio; HR: 4.06, *p* < 0.0001) and HAQ at baseline (HR: 2.42, *p* = 0.0024) significantly increased the risk of SB4 discontinuation, while previous ETA duration was negatively associated with SB4 discontinuation (HR: 0.97, *p* = 0.0064). Forty-one (17.4%) patients left the study due to the interruption of the SB4 treatment, 31 (75.6%) discontinued due to inefficacy and 10 (24.4%) due to adverse events. This real-life study confirms the similar efficacy profile of ETA with long-term retention and a good safety profile in inflammatory arthritis patients.

## 1. Introduction

Rheumatoid arthritis (RA), spondyloarthritis (including ankylosing spondylitis; AS) and psoriatic arthritis (PsA) are chronic inflammatory rheumatic diseases characterised by different clinical, laboratory and imaging hallmarks [1,2,3] that negatively impact upon patient quality of life. The prevalence of these disorders varies depending on genetic and environmental factors [4] and is estimated to affect between 0.1–1% of individuals worldwide [5,6,7]. Over the past two decades, biological drugs have played a central role in the treatment of RA, AS and PsA due to their effectiveness in reducing signs and symptoms of the disease over both the short and long term. However, biologic drugs can also be associated with the presence of adverse events not related to their specific mechanism of action, that are most frequently associated with infections, musculoskeletal and skin disorders [8,9].

Among biological disease modifying anti-rheumatic drugs, (bDMARDs), inhibitors targeting tumour necrosis factor (TNF) alpha were the first to achieve long-term remission and to significantly improve patients’ quality of life [10]. Despite their elevated cost, the three licensed TNF inhibitors (adalimumab, infliximab and etanercept) are still ranked among the most frequently used biologic drugs for the treatment of RA and PsA [11].

In recent years, many biologics, including anti TNF-alpha biologics (Remicade/Infliximab, Enbrel/Etanercept, Humira/Adalimumab) have expired, leading to the rapid development and availability of more affordable biosimilars. A biosimilar is a biological drug that is highly similar, with regard to its clinical behaviour (including pharmacokinetics, efficacy, safety and immunogenicity) to a previously approved and existing biologic treatment (the originator or reference product) [12].

The ETA biosimilar SB4 (Benepali) was recently developed [13,14], with equivalent clinical efficacy demonstrated in phase III randomised clinical trials in RA patients [15,16,17]. Compared with non-switched patients treated with SB4 or ETA, no difference was observed in those patients switched from ETA to SB4 [17]. In terms of safety, in another phase III clinical trial involving RA patients, SB4 was shown to be less associated with injection site reactions and to have less immunogenic power than ETA [18].

In the large real-life DANBIO cohort that examined non-medical switch in inflammatory arthritis patients, the retention rate was found to be higher than ETA non-switched patients but lower than the historical ETA cohort [19]. These findings were also confirmed in other real-life cohorts, where SB4 and ETA originators showed similar effectiveness in maintaining low disease activity in PsA patients, as well as in plaque psoriasis patients without arthritis [20,21,22,23].

In Italy, the Italian drugs agency (AIFA-Agenzia Italiana del Farmaco) published a position paper in 2018 on biosimilars and switching [24], encouraging physicians to strongly consider literature on the safety and efficacy of the switch, reminding the physicians of their role and responsibility in the economic sustainability of the health system. In the Piedmont Region in North Italy, following AIFA approval for the reimbursement of SB4, the prescription of biosimilars is recommended for drug-naïve patients that require a specific target therapy, whilst the switch from originators to biosimilars is encouraged for all patients treated with the originator [25]. Regional recommendations refer exclusively to ETA and do not interfere with the possibility of prescribing the most suitable bDMARD or tsDMARD. The aim of this real-life study was to evaluate disease activity and persistence of SB4 after switching from ETA to its biosimilar SB4 in patients with stable inflammatory arthritis over a period of 3 years.

## 2. Materials and Methods

### 2.1. Patients

In this real-life study, we selected patients with clinical diagnosis of rheumatoid arthritis (RA), psoriatic arthritis (PsA) and ankylosing spondylitis (AS) from eight rheumatology units in Italy. Patients had been previously treated with etanercept (ETA) Enbrel^®^ and switched to the ETA biosimilar SB4. As suggested by regional documentation, off-label, pregnant and paediatric patients, patients with a history of allergy, patients not in remission nor in low disease activity and patients with psychological reasons that would not allow for a change in drug, were excluded [25]. As per EULAR guidelines [26], we also excluded patients who refused the switch. At the time of the switch, every patient was informed about biosimilar properties, literature data and the possibility to return to the originator if necessary [26,27]. Almost all patients accepted the switch.

### 2.2. Outcome Measures

At each outpatient visit, we recorded demographic features (age, sex and time since RA, PsA or AS diagnosis) and the following disease activity measures: Disease Activity Score in 28 joints (DAS28) [28], Disease Activity for Psoriatic Arthritis score (DAPSA), Bath Ankylosing Spondylitis Disease Activity Index (BASDAI) [29] and the Health Assessment Questionnaire (HAQ) [30]. For peripheral joint assessment, 68 joints were assessed for tenderness and 66 joints were assessed for swelling. Rheumatoid factor (RF), anti-citrullinated protein antibody (ACPA), C-reactive protein (CRP) and human leukocyte antigen B27 (HLAB27) were also measured over the observational period (visits 0, 12, 24 and 36 months).

Data were stratified by age, sex, duration of disease and concomitant therapy. The disease activity was evaluated during the year before the introduction of the SB4, and then evaluated in the following 36 months during ETA treatment. We also examined whether some baseline characteristics, such as the duration of ETA treatment, concomitant therapy (conventional synthetic DMARDs and glucocorticoids) presence of comorbid disease and baseline disease activity, could influence the SB4 discontinuation. Written informed consent for the anonymous use of personal data was obtained from every patient, in compliance with Legislative Decree 196/2003. Approval was obtained from the local ethics committee (AOU Citta’ della Salute e Della Scienza di Torino—AO Mauriziano—ASL TO1; protocol number: 0127142, approved on 29 January 2015) for this study. This study complies with the ethical standards laid down in the 1975 Declaration of Helsinki.

### 2.3. Statistical Analysis

Data are presented as mean ± standard deviation for continuous variables, median and interquartile range (IQR) in case of not normally-distributed variables, and count (%) for categorical data. Non-parametric and parametric tests (Kruskal–Wallis test, Mann–Whitney U test and χ2 test) were used to compare sample characteristics. Survival distribution curves were computed by the Kaplan–Meier method. Stepwise Cox proportional hazard multivariate models were developed to examine potential predictors of SB4 withdrawal in RA, PsA and AS patients.

Results are presented as hazard ratios (HRs) with 95% confidence intervals (95% CI). A *p*-value ≤ 0.05 was considered statistically significant. Statistical analyses were performed using MedCalc Statistical Software version 18.2.1 (MedCalc Software Bvba, Ostend, Belgium) and R Foundation for Statistical Computing, Vienna, Austria, version 4.0.

## 3. Results

### 3.1. Baseline Clinical Characteristics

A total of 236 patients were included in this real-life observational study: 120 (50.8%) in the RA group, 80 (33.9%) in the PsA group and 36 (15.3%) in the AS group. Baseline clinical characteristics of patient groups are summarised in Table 1. The majority of patients were female in both RA (*n* = 97, 80.8%) and PsA groups (*n* = 44, 55%) with a higher proportion of male patients affected with AS (*n* = 26, 72.2%). Mean age was slightly higher in RA and PsA patients (61.8 ± 14.6 years and 61.8 ± 12.8 years, respectively) compared to the AS group (54.9 ± 11.7 years) and disease duration was longer in RA patients (17.2 ± 10.6) compared to PsA (14.2 ± 7.2) and AS patients (14.9 ± 9.5). The most frequent comorbid diseases were diabetes (5.5%), chronic bronchitis (2.5%) and cerebrovascular disease (2.1%). Although CRP levels were higher in RA patients compared to PsA and AS patients, most patients presented with stable, low grade disease activity, as observed by DAS28 (2.5 ± 0.75) for RA, DAPSA (3.7 ± 2.7) for PsA patients and BASDAI scores (1.5 ± 1.6). Almost half (47.5%) of RA patients were receiving concomitant corticosteroid (prednisone) treatment compared to 28.7% of PsA and 13.9% of AS patients. RA and AS patients were previously treated with ETA for a longer period and received SB4 for a longer duration compared to PsA patients.

### 3.2. Drug Survival

**Figure 1:**
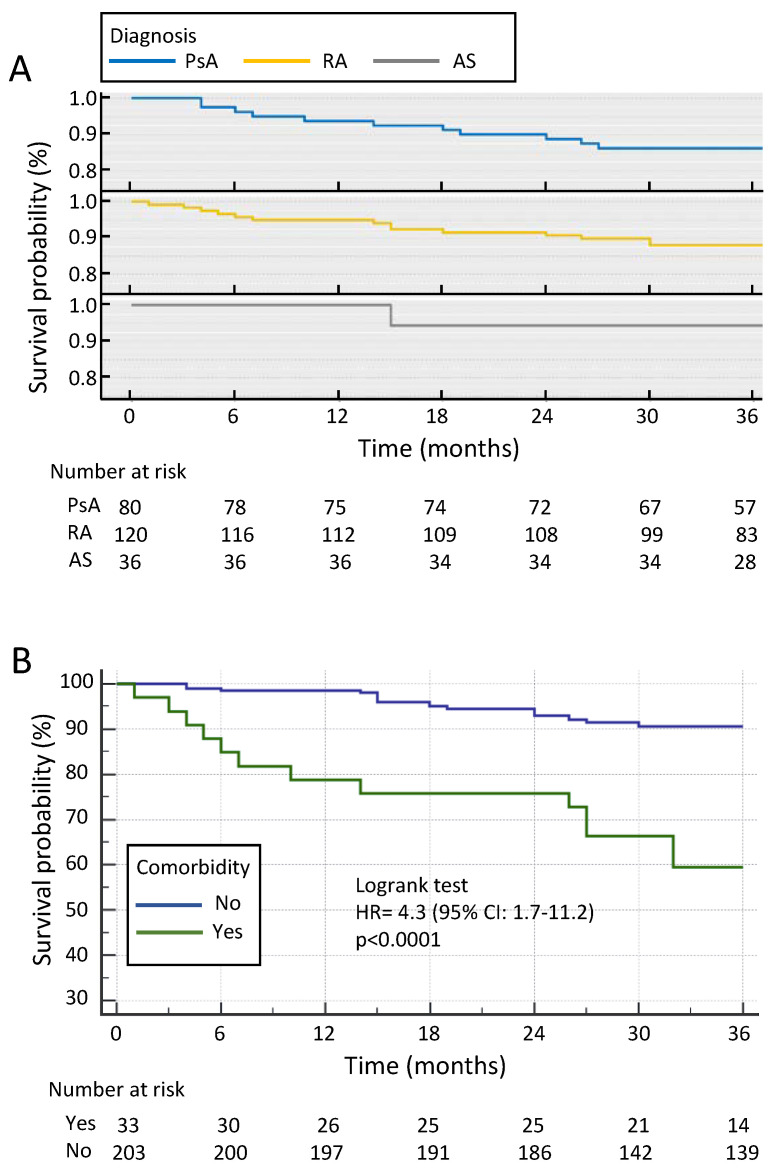
Kaplan–Meier curve showing retention rate in RA, PsA and AS patients over 3 years treated with SB4 after ETA failure. (**A**) Cumulative retention probability in RA, PsA and AS patients; (**B**) retention rate in all patients with and without comorbidity. AS = ankylosing spondylitis; ETA = etanercept; PsA = psoriatic arthritis; RA = rheumatoid arthritis.

Although overall drug survival was slightly favourable in AS patients, no significant difference was observed compared to RA patients (HR 4.8, 95% CI: 1.9–11.7; *p* = 0.07) or PsA patients (HR 3.5, 95% CI: 1.5–8.4; *p* = 0.07) (Figure 1A). The 3-year retention rate of SB4 was 94.4% (95% CI: 87–100%) in AS patients, 88% in RA patients (95% CI: 82–94%) and 86% in PsA patients (95% CI: 79–94%). Patients stratified for the presence of comorbid disease revealed a significantly higher retention rate after 3 years in patients without comorbid disease vs. those burdened with a comorbid disease (90% vs. 60%, HR 4.3, 95% CI: 1.7–11.2; *p* < 0.0001) (Figure 1B).

### 3.3. Effectiveness of SB4

**Figure 2:**
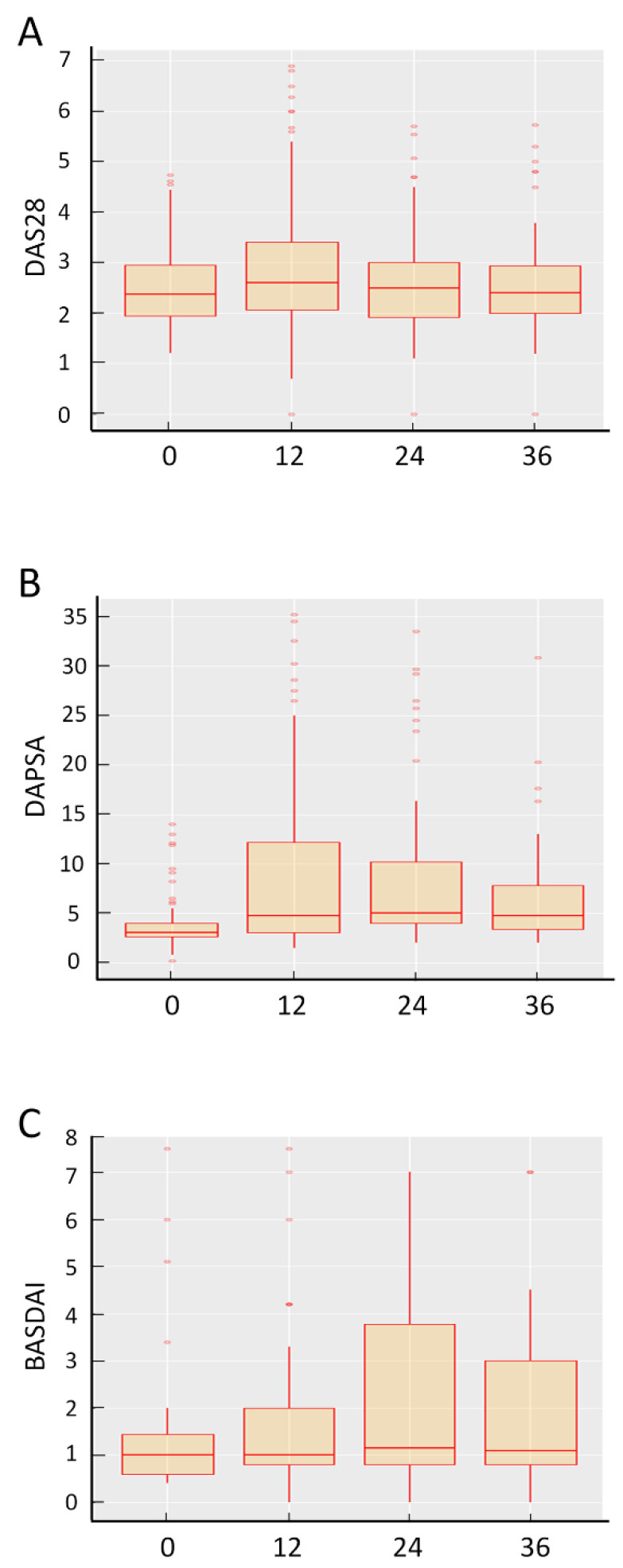
Box and whisker plots illustrating changes in disease activity for the different pathologies (RA, PsA and AS) over the follow up period. (**A**) DAS 28 was measured in RA patients, (**B**) DAPSA in PsA patients and (**C**) BASDAI in AS patients, respectively, at baseline and after 3 years of treatment with SB4. Data presented as median, 25th/75th percentiles and maximum/minimum recorded values. Orange open dots represent standard outliers (fall between 1.5 × IQR and 3.0 × IQR outside of the IQR,) whereas red full dots represent extreme outliers (fall greater than 3.0 × IQR outside the IQR).

Disease activity for the different pathologies (RA, PsA and AS) with their respective clinimetric indices (DAS28, DAPSA and BASDAI) were examined over the follow-up period. In RA patients, DAS28 values remained unchanged over 3 years with a median of 2.37 (IQR 1.9–2.9) at baseline vs. 2.5 (2–3) after 3 years (Figure 2). Likewise, in AS patients, BASDAI remained unchanged after 3 years compared to baseline values (1; 0.6–1.5 vs. 1.05; 0.8–2.25) (Figure 2). Although a statistically significant increase was detected for DAPSA in PsA patients (3.05; 2.7–4.0 vs. 4.55; 3.2–7.5, *p* < 0.001), this difference was not judged as being clinically significant (Figure 2).

### 3.4. Predictors of SB4 Discontinuation

Multivariate regression models were next used to examine predictors of SB4 discontinuation in patients over the follow up period. In all the models studied, the variables considered were age, gender, body mass index (BMI), disease duration, duration of ETA, HAQ, seropositivity (RF and ACPA), line of therapy, combination therapy, steroid, comorbidity (Charlson Comorbidity Index, CCI), remission or low disease activity and HLAB27 positivity (only for PsA or AS).

**Figure 3:**
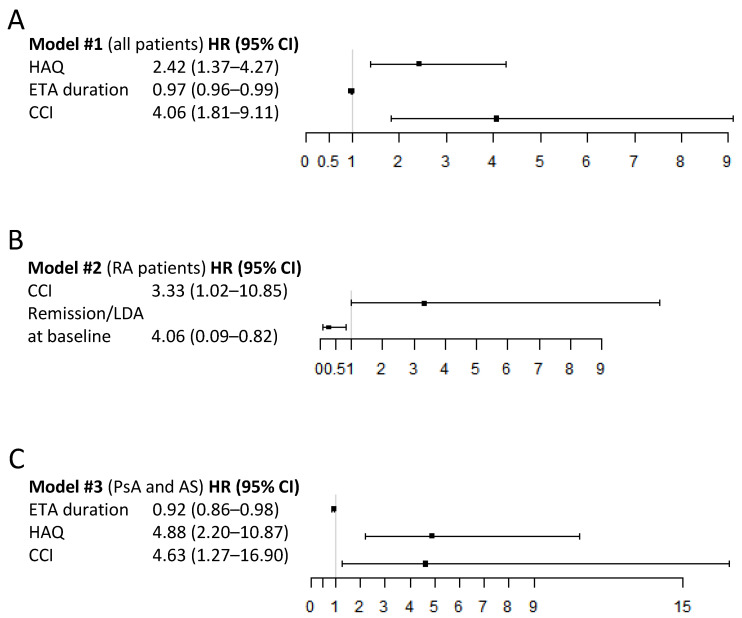
Forest plots showing predictive variables of maintaining the response after switch from ETA to SB4. (**A**), Model#1 including all patients, (**B**), Model#2 stratified for RA patients and (**C**), Model#3 in patients who were seronegative (i.e., PsA and AS patients). Data presented as hazard ratio (HR) and 95% confidence intervals (CI). CCI = Charlson Comorbidity Index; HAQ = Health Assessment Questionnaire; PsA = psoriatic arthritis; RA = rheumatoid arthritis.

In a first model including all patients, the presence of comorbid disease (HR 4.06, 95% CI: 1.81–9.11, *p* = 0.0007, Figure 3A) and HAQ at baseline (HR 2.42, 95% CI: 1.37–4.27, *p* = 0.0024, Figure 3A) significantly increased the risk of SB4 discontinuation while previous ETA duration was negatively associated with SB4 discontinuation (HR 0.97, 95% CI: 0.96–0.99, *p* = 0.0064, Figure 3A).

In a second model stratified for RA patients, the presence of comorbid disease (HR 3.33, 95% CI: 1.02–10.85, *p* = 0.0046, Figure 3B) was significantly associated with SB4 discontinuation. Otherwise, remission or LDA at baseline proved to be a protective factor (HR 0.27, 95% CI: 0.09–0.82, *p* = 0.02, Figure 3B). Seropositivity, understood as rheumatoid factor and/or ACPA positivity, did not show any correlation.

In a third model in patients who were seronegative (i.e., PsA and AS patients), HAQ score (HR 4.88, 95% CI: 2.20–10.87, *p* = 0.0001, Figure 3C) and comorbid disease (HR 4.63, 95% CI: 1.27–16.90, *p* = 0.021, Figure 3C) were all significantly associated with SB4 discontinuation, while duration of ETA at baseline (HR 0.92, 95% CI: 0.86–0.98, *p* = 0.007, Figure 3C) was favourable in maintaining biosimilar therapy.

### 3.5. Reasons for Discontinuation

**Figure 4:**
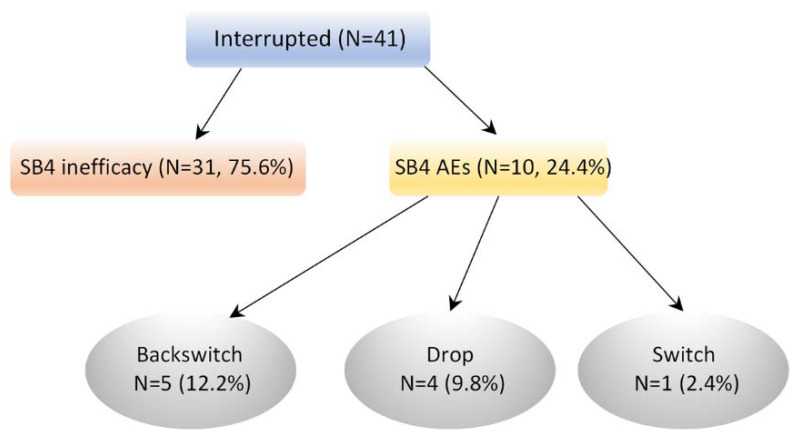
Flow diagram showing the number of patients discontinuing SB4 treatment after receiving ETA.

A total of 41 patients interrupted treatment with SB4 and the majority of patients stopped treatment due to inefficacy (*n* = 31, 75.6%) (Figure 4). Ten (24.4%) patients stopped treatment due the presence of an adverse event of which half of these were switched back to ETA, four dropped out of treatment and one switched to another biological treatment.

## 4. Discussion

Findings from this 3-year real-life study involving 236 patients with RA, PsA and AS show that switching from ETA to SB4 allowed patients to achieve a high rate of persistence with a low rate of adverse events. The 3-year retention rate for SB4 was 94% in AS patients, 86% in RA patients and 83% in PsA patients with no difference between patients. Of the 41 (17.4%) patients who discontinued treatment, 31 (13.1%) were due to inefficacy and 10 (4.2%) due to the presence of an adverse event. Disease activity measures also remained stable over the 3-year follow-up period.

Our results corroborate those from other real-life studies examining drug persistence and safety after switching from ETA to SB4 in patients with inflammatory rheumatic diseases.

In another real-life study performed in Italy, Bruni et al. recently evaluated the persistence and safety of SB4 after switching from ETA in 220 patients with clinically stable inflammatory arthritis, comprising RA, PsA and axSpA [23]. Patient characteristics were similar to those in our study in terms of age (58 years) and disease duration (14 years); they observed a cumulative probability of persistence for SB4 of 99.1, 88.6 and 64.6% at 6, 12 and 18 months, respectively. Persistence was considerably higher in our population at these same time points (95–97% between 6–12 months and >90% at 18 months). This may be attributed to the higher incidence of adverse events (22.7% vs. 4.2%) and longer previous ETA duration (7 vs. 4.1 years in our study, a factor that was negatively associated with discontinuation in our analysis), although only age emerged as a predictor of SB4 discontinuation in their Cox regression analysis. In contrast, the presence of comorbid disease and low disease activity/remission at baseline emerged as predictor of SB4 discontinuation in our analysis. In fact, patients stratified for the presence of comorbid disease had a significantly lower retention rate (60% vs. 90%, *p* < 0.0007), up to 3 years. The presence of comorbidities would be expected to negatively impact upon drug adherence since these patients tend to have a higher rate of treatment interruption due to complications [31,32,33]. When we stratified patients by disease type, the presence of comorbid disease and elevated HAQ value (<2) was associated with SB4 discontinuation. However, some differences did emerge. In RA patients, remission or LDA is a factor favourable to the maintenance of biosimilar therapy. This phenomenon can be explained by the fact that in RA, it is possible to achieve remission through the “treat to target” strategy in an easier way than in SpA, so the patient is more stable from the point of view of disease activity [34].

In PsA and AS patients (seronegative), HAQ score was also associated with SB4 discontinuation, indicating that greater disability (denoted by higher HAQ score) negatively impacts on the adherence. Patients with more active disease may be harder to treat and thus may be more depressed [35] and more likely to discontinue treatment [35,36,37].

In the large DANBIO registry, 1621 patients (933 RA, 351 PsA and 337 axSpA) switched from ETA to SB4 and the 1-year (crude) persistence to SB4 was 82% compared to 88% in an ETA historic cohort and 70% in the non-switched population [19]. A total of 299 (18.4%) patients discontinued SB4 and 137 (8.4%) were due to lack of efficacy and 77 patients (4.8%) due to adverse events. Although SB4 persistence was higher in our cohort (95–100%), discontinuation rates were similar. In our study population, 41 (17.4%) patients who discontinued SB4 were due to loss of efficacy or adverse events in 31 (13.1%) and 10 (4.2%) patients, respectively, almost identical to those based on the DANBIO registry.

In the Bio-SPAN study performed in the Netherlands, 625 patients (433 RA, 128 PsA and 64 AS) were switched from ETA to SB4; the cumulative 6-months persistence rates for SB4 and ETA were 90% vs. 92%, respectively, with discontinuation rates of 10% at 6 months [38]. In another 6-month real life study conducted across four European countries, 358 patients with RA and 199 patients with axSpA were switched from ETA to SB4 [39]. The 6-month retention rate was 90.8% in the RA group and 92.4% in the axSpA cohort, similar to the Bio-SPAN study. However, 31 (5.6%) patients reported adverse events, seven (1.3%) regarded as serious.

In a systematic review by Ebbers et al. including 959 articles and a total of 13,552 patients, retention rates across studies were around 75% at 12 months, lower than values observed in our cohort at 12 months and even up to 3 years [40]. These differences in retention rates may be attributed to heterogeneity in patients included in the systematic review. In addition, evidence was mainly derived from congress abstracts/editorials (25/31 publications; 80.6%) and the quality was generally lower with greater variability in these publications compared to full journal papers [40].

Besides persistence and efficacy data, other evidence is available showing the economic benefit from switching patients to biosimilars that could allow for more efficient allocation of health care resources and therefore improving patient care. Shah and colleagues performed a cost analysis on 151 patients switched from ETA to SB4 in a UK hospital. They estimated that the switch (between January 2017 and June 2017) saved approximately GBP 500,000 per annum [41].

Analysis of our real-life data in those patients who agreed to switch confirms results derived from trials and real-world data. In particular, there were no statistically significant differences in disease activity after the switch to SB4 and during the follow up (3 years after the switch). Furthermore, no significant differences were observed in the high rates of persistence among patients with RA, PsA, or AS. Adverse events were not serious. Among the available evidence on real-life data, this is the first evidence with up to 3 years of follow-up.

## 5. Study Limitations

Nonetheless, this descriptive study has some limitations that must be highlighted. The main limitation of this real-life study was the small sample size and the unequal number of patients affected by the three rheumatic diseases. Despite this, clinical characteristics, (particularly disease severity), were comparable among the groups permitting pooled analysis, such as drug survival and Cox regression analysis, also confirmed by the little variation in retention rate across the three groups over 3 years. However, for some features, such as the presence of comorbid diseases, that was mainly seen in RA and PsA patients and only accounted for a total of 33 patients; caution needs to be taken when interpreting some of these sub-analyses. No data are available on drug pharmacokinetics or levels of anti-drug antibodies, from either the originator or the biosimilar. The nocebo effect was not examined using psychometric measures.

## 6. Conclusions

This study provides real-world evidence on the 3-year persistence and efficacy of the biosimilar SB4 after switching from the originator ETA. Persistence was maintained over 90% across the three patient cohorts while disease activity remained stable over the 3 years with only 17.4% of patients discontinuing (13.1% due to inefficacy and 4.2% due to adverse events). Although only a small proportion of patients were burdened with comorbid diseases, these patients showed a higher rate of discontinuation and as such should receive particular attention and tailored treatment in order to manage potential complications that lead to discontinuation. In the absence of studies with a larger sample size and longer follow-up period, these real-world data provide the best available evidence to aid rheumatologists in the therapeutic management of these patients.

## Figures and Tables

**Table 1:** Baseline characteristics of RA, PsA and AS patients.

Characteristics	Total Cohort *n* = 236	RA *n* = 120	PsA *n* = 80	AS *n* = 36
*n* (%)	236 (100)	120 (50.8)	80 (33.9)	36 (15.3)
Male gender (%)	85 (36)	23 (19.2)	36 (45)	26 (72.2)
Age (years)	60.7 ± 13.8	61.8 ± 14.6	61.8 ± 12.8	54.9 ± 11.7
BMI (Kg/M^2^)	24.5 ± 4.2	24 ± 3.8	24.5 ± 3.9	24.7 ± 3.9
Disease duration (years)	15.9 ± 9.5	17.2 ± 10.6	13.8 ± 6.6	14.9 ± 9.5
Comorbidities, *n* (%)	33 (14)	17 (14)	15 (19)	1 (3)
Diabetes	13 (5.5)	7 (5.8)	6 (7.5)	-
Chronic bronchitis	6 (2.5)	3 (2.5)	3 (3.8)	-
Cerebrovascular disease	5 (2.1)	1 (0.83)	3 (3.8)	1 (2.8)
Liver disease	2 (0.85)	2 (1.7)	-	-
Myocardial infarction	2 (0.85)	2 (1.7)		
Heart failure	1 (0.42)	1 (0.83)		
Renal failure	1 (0.42)	1 (0.83)		-
Connectivitis	2 (0.85)	-	2 (2.5)	-
Peptic ulcer	1 (0.42)		1 (1.3)	
ACPA/RF (+/+), *n* (%)	-	76 (63.3)	-	-
RF+, *n* (%)	-	75 (62.5)	-	-
HLAB27+, *n* (%)	-	-	12 (15)	18 (50)
TJC (median (IQR)	0 (0–1)	0 (0–2)	0 (0–1)	0 (0–0)
SJC (median (IQR)	0 (0–0)	0 (0–1)	0 (0–0)	0 (0–0)
CRP (mg/L)	1.7 ± 2.3	2.2 ± 1.9	1.3 ± 2.8	1.3 ± 1.9
DAS 28	-	2.5 ± 0.7	-	-
DAPSA	-	-	3.7 ± 2.7	-
BASDAI	-	-	-	1.5 ± 1.6
HAQ	0.7 ± 0.6	0.7 ± 0.5	0.7 ± 0.6	0.8 ± 0.7
Medication				
Combination therapy, *n* (%)	132 (55.9)	77 (64.2)	43 (53.8)	12 (33.3)
Prednisone, *n* (%)	85 (36)	57 (47.5)	23 (28.7)	5 (13.9)
Etanercept duration (months)	49.74 ± 40.75	56.43 ± 41.27	25.00 ± 13.67	40.08 ± 37.78
SB4 duration (months)	38.42 ± 11.41	37.77 ± 11.84	13.72 ± 13.49	41.39 ± 8.95

Data are reported as mean ± standard deviation, frequencies (number and %) or median and interquartile range (IQR). ACPA/RF = anti–citrullinated protein antibody/rheumatoid factor; AS = ankylosing spondylitis; BASDAI = Bath Ankylosing Spondylitis Disease Activity Index; BMI = body mass index; CRP = C-reactive protein; DAS28 = Disease Activity Score 28; DAPSA = Disease Activity in Psoriatic Arthritis; HAQ = Health Assessment Questionnaire; HLAB27 = human leukocyte antigen B27; PsA = psoriatic arthritis; RA = rheumatoid arthritis; SB4 (etanercept biosimilar, Benepali^®^); SJC = swollen joint count; TJC = tender joint count.

## Data Availability

Data can be made available from the corresponding author upon request.
